# Influence of family size and birth order on risk of cancer: a population-based study

**DOI:** 10.1186/1471-2407-11-163

**Published:** 2011-05-09

**Authors:** Melanie Bevier, Marianne Weires, Hauke Thomsen, Jan Sundquist, Kari Hemminki

**Affiliations:** 1 Division of Molecular Genetic Epidemiology, German Cancer Research Center (DKFZ), Im Neuenheimer Feld 580, D-69120 Heidelberg, Germany; 2 Center for Primary Health Care Research, Lund University, Malmö, Sweden; 3 Stanford Prevention Research Center, Stanford University School of Medicine, California, USA

## Abstract

**Background:**

Family size and birth order are known to influence the risk of some cancers. However, it is still unknown whether these effects change from early to later adulthood. We used the data of the Swedish Family-Cancer Database to further analyze these effects.

**Methods:**

We selected over 5.7 million offspring with identified parents but no parental cancer. We estimated the effect of birth order and family size by Poisson regression adjusted for age, sex, period, region and socioeconomic status. We divided the age at diagnosis in two groups, below and over 50 years, to identify the effect of family size and birth order for different age periods.

**Results:**

Negative associations for increasing birth order were found for endometrial, testicular, skin, thyroid and connective tissue cancers and melanoma. In contrast, we observed positive association between birth order and lung, male and female genital cancers. Family size was associated with decreasing risk for endometrial and testicular cancers, melanoma and squamous cell carcinoma; risk was increased for leukemia and nervous system cancer. The effect of birth order decreased for lung and endometrial cancer from age at diagnosis below to over 50 years. Combined effects for birth order and family size were marginally significant for thyroid gland tumors. Especially, the relative risk for follicular thyroid gland tumors was significantly decreased for increasing birth order.

**Conclusion:**

Our findings suggest that the effect of birth order decreases from early to late adulthood for lung and endometrial cancer.

## Background

Family size and birth order has been shown to have an effect on the risk of cancer through socioeconomic and biological factors. For example, local observations showed that lower birth weight as a consequence of higher birth order has been associated with a lower risk for breast cancer [1-3] and a lower risk for melanoma [2]. Negative association has also been detected in testicular cancer for children of higher birth order [4-6]. Higher birth order often implies higher parental age at conception, although the latter has not been reported to be a risk factor in some studies [7,8] whereas in other studies there was an association found for cancer sites as breast and prostate cancer as well as childhood cancers [9-13]. Genetic diseases or cancer during childhood may shorten the reproduction phase of parents, which could cause higher risk for individuals in families with fewer children [14]. This might lead to an association of early-onset cancer or childhood cancers within last born children. Risks for many types of cancer and morbidities have been associated with the socioeconomic status of an individual [15,16]. A decreasing risk for melanoma has been reported for increasing family size and was explained by limited affordability of sunny holidays and solarium visits of larger families [14,17,18]. Significant association between socioeconomic factors, family size and lung cancer has also been reported in a previous study based on the Swedish Family-Cancer Database [19,20]. Socioeconomic factors have been shown to influence obesity which is an important risk factor for endometrial and other cancers such as liver cancer, non-Hodgkin lymphoma and multiple myeloma [21-24]. The susceptibility to be overweight might be influenced by childhood environment and to be carried on to adulthood. Closer contact among family members of larger families has been shown to increase risk of infection with Helicobacter pylori and other Helicobacter pylori related cancers [2,25,26].

The goal of the present study was to systematically analyze the effects of birth order and family size on different types of cancer. As we used the newest update of the Swedish Family Cancer-Database we had more than 75,000 additional registered cancer cases as previous studies with 178,365 cases [19]. This updated version included a total of 254,697 of cancer cases in offspring. Excluding the offspring with affected parents resulted in a study population of 134,896 individuals. The larger number of cancer cases leaded to more robust estimates of associations and allowed us to include also more rare cancers, such as esophageal, eye, small intestinal, laryngeal and salivary gland cancers. Additionally, we were able to divide the age at diagnosis in two categories to quantify the effect of family size and birth order. Differences in risk estimates for individuals diagnosed before and after 50 years of age are useful for identifying the effects of birth order and family size during life. Both influence the childhood environmental and lifestyle. Our aim was to see whether these factors still have an influence on cancer during early and later adulthood and whether they change over time. We focused on that aspect, as this has not been analyzed in previous studies.

## Methods

The Swedish Family-Cancer Database includes data from the Second-Generation Register, the Swedish Cancer Registry, the National Census and the Death Notification Registry covering all cancers from 1961 to 2006 according to the seventh revision of the International Classification of Diseases (ICD-7) [27]. Cancer studies (Family-Cancer Database) in the MigMed Database was approved by the Lund regional ethical committee on 8/12/2008 (No. 409/2008) and with complementary approvals dated 9/1/2009 and 1/22/2010. A total of approximately 11 million individuals born after 1932 and their biological parents have been registered. The newest version assembled in 2009 contains also information on more than 1.2 million first and multiple primary cancers and in situ tumors [25]. For most of the individuals affected with cancer clinical information on tumors is also available. Additionally, residential and socioeconomic data are available from national censuses, which were carried out in 1960, 1970, 1980 and 1990.

The study included all individuals from the database with identified parents. Individuals having parents affected with cancer were excluded, because parental cancer history should not be taken into account to avoid any confounding effect. The risk through parental cancer cases will be separated from the risk caused by birth order or family size. This lead to a study population of 5,657,455 individuals where 134,896 individuals were affected with cancer. The birth order of every individual was defined through the mother's parity (grouped 1, 2, 3-4, 5-17). The family size (grouped 1, 2, 3-4, 5-17) is defined as the number of children per mother. There were 669,137 singletons, more than 2.3 million offspring in families with two children, more than 2.2 million in families with three or four children and 437,907offspring in families with at least five children included in the study. To analyze the effects of birth order and family size for early and later adulthood the individuals were categorized for age at diagnosis less and over age 50.

A four-digit diagnostic code according to ICD-7 was used to identify cancer sites. Some cancers were grouped according to the following codes: upper aerodigestive tract (140-141.9, 143.0-148.9), rectum excluding anus (154-154.0, 154.8), liver and gallbladder (155.0-156.9), lung (162.0-163), uterus (173-174), non-Hodgkin lymphoma (200-200.2, 202-202.2) and leukemia (204.0-209.9).

Patients were followed up for a specific time interval depending on the information available for each person in the database. Follow up started at year of immigration, birth year, or start year of cancer registry (1961), whatever came latest, until year of diagnosis, death, emigration or end of the study (2006), whatever occurred first. Cancer cases and person-years were determined for every stratification class of the covariates included in the regression model. For the calculation of the relative risks and the corresponding 95% confidence intervals, age at stopfollow (5-year-bands), sex, calendar period (1961-1985, 1986-1990, 1991-1995, 1996-2000, 2001-2006), region (big cities, northern Sweden, southern Sweden, other), socioeconomic status (agricultural worker, white-collar worker, other worker, professional, private, other), family size, and birth order have been included as covariates. The Genmod procedure in SAS (SAS version 9.2; SAS Institute, Cary, NC, USA) was used to fit the Poisson regression model. The cancer status (affected or not affected) is specified as a response variable, whereas the included covariates are explanatory variables. Parameter estimates (β) are obtained by maximum likelihood estimation (MLE). These parameters are estimated numerically through an iterative fitting process. Cancer incidence for one group compared to the reference group was calculated to obtain relative risk estimates. P values for trend analysis were calculated using a Jonckheere-Terpstra test which is a nonparametric test for ordered differences among classes.

## Results

The study included around 5.7 million individuals, of which 134,896 were affected with cancer. Table 1 gives an overview on the distribution of individuals and cancer cases by family size and birth order. In Table 2 the age at stop follow of the individuals included in the study is presented.

**Table 1:** Number of all individuals (and cancer cases) included in the study with respect to birth order and family size

All families			Family size		
Birth order	1	2	3-4	5-17	All
1	669,137 (25,209)	1,161,073 (26,279)	681,658 (20,129)	75,738 (4,226)	2,587,606 (75,843)
2		1,169265 (18,325)	686,860 (15,439)	76,402 (3,543)	1,932,527 (37,307)
3-4			851,555 (13,395)	154,668 (5,315)	1,006,223 (18,170)
5-17				131,099 (3,036)	131,099 (3,036)
All	669,137 (25,209)	2,330,338 (44,604)	2,220,073 (48,963)	437,907 (16,120)	5,657,455 (134,896)

**Table 2:** Age at stopfollow of individuals (and cancer cases) included in the study

Age at stopfollow	All individuals	Cancer cases (%)
0-4	551,205	3,405 (0.62)
5-9	508,742	2,277 (0.45)
10-14	566,083	2,017 (0.36)
15-19	585,185	2,753 (0.47)
20-24	493,490	3,728 (0,76)
25-29	454,454	5,161 (1.14)
30-34	446,358	6,537 (1.46)
35-39	411,925	8,014 (1.95)
40-44	376,844	12,213 (2.71)
45-49	296,309	13,364 (4.51)
50-54	273,077	17,052 (6.24)
55-59	276,905	20,842 (7.53)
60-64	264,047	20,190 (7.65)
65-69	178,049	14,516 (8.15)
70-	109,678	4,827 (4.40)
All	5,657,455	134,896 (2.33)

Table 3 shows relative risk estimates for birth order of all cancer sites analyzed separately for age at diagnosis below 50 years or above 50 years and combining both. Significant results at 5% confidence level are written bold. A significantly increased relative risk for lung cancer (RR = 1.08, 99% CI: 1.00-1.15) was found independent of the age at diagnosis. Birth order was associated with a decreased risk for endometrial and testicular cancers, melanoma, squamous cell skin cancer, and cancers of the thyroid gland and connective tissue. The relative risk for cervical cancer (RR = 0.83) was significantly decreased for individuals diagnosed below age 50 years.

**Table 3:** Relative risks for birth order and age at diagnosis

		Age at diagnosis < 50 years			Age at diagnosis ≥ 50 years			All		
			Birth order	Ref		Birth order	Ref		Birth order	Ref
Cancer site	(ICD-7 code)	N	RR (95% CI)	N	N	RR (95% CI)	N	N	RR (95% CI)	N
Upper aero-digestive tract	(140+)	402	0.95 (0.82-1.09)	396	577	1.09 (0.98-1.21)	807	979	1.04 (0.95-1.14)	1203

Salivary glands	(142)	119	**0.79** (0.63-0.99)	141	59	0.93 (0.65-1.33)	96	178	0.85 (0.70-1.04)	237

Esophagus	(150)	55	0.89 (0.59-1.37)	52	248	0.89 (0.75-1.05)	435	303	0.89 (0.76-1.05)	487

Stomach	(151)	261	1.03 (0.84-1.26)	232	505	1.07 (0.95-1.19)	757	766	1.06 (0.94-1.20)	989

Small intestine	(152)	84	0.94 (0.71-1.24)	85	147	1.12 (0.92-1.36)	206	231	1.07 (0.91-1.26)	291

Colon	(153)	938	0.94 (0.85-1.04)	928	1532	0.97 (0.91-1.04)	2723	2470	0.97 (0.91-1.02)	3651

Rectum	(154/1541)	376	1.03 (0.85-1.26)	343	1084	1.03 (0.96-1.11)	1740	1460	1.03 (0.95-1.12)	2083

Anus	(1541)	66	1.08 (0.77-1.50)	56	85	0.95 (0.74-1.24)	133	151	1.02 (0.83-1.24)	189

Liver and gallbladder	(155, 156)	217	0.95 (0.80-1.14)	202	467	0.92 (0.82-1.04)	807	684	0.94 (0.85-1.04)	1009

Pancreas	(157)	134	0.88 (0.69-1.12)	128	573	0.93 (0.81-1.07)	972	707	0.93 (0.84-1.03)	1100

Nose	(160)	49	0.99 (0.64-1.52)	46	47	1.19 (0.85-1.67)	67	96	1.05 (0.76-1.45)	113

Larynx	(161)	58	0.82 (0.61-1.10)	71	153	1.05 (0.83-1.32)	227	211	1.00 (0.83-1.19)	298

Lung	(162, 163)	589	**1.17** (1.04-1.31)	454	2201	1.03 (0.97-1.09)	3343	2790	**1.08** (1.02-1.13)	3797

Breast	(170)	5053	1.03 (0.82-1.28)	4778	5937	0.98 (0.95-1.01)	8751	10990	1.00 (0.91-1.11)	13529

Cervix	(171)	1609	0.95 (0.85-1.06)	1597	245	**0.83** (0.71-0.97)	401	1854	0.93 (0.85-1.02)	1998

Endometrium	(172)	213	**0.72** (0.64-0.81)	1042	321	**0.87** (0.83-0.92)	1924	1255	**0.85** (0.81-0.90)	2245

Uterus	(173, 174)	140	0.91 (0.79-1.04)	151	106	0.99 (0.82-1.19)	166	246	0.95 (0.85-1.06)	317

Ovary	(175)	902	0.98 (0.88-1.08)	868	806	1.03 (0.95-1.13)	1177	1708	1.01 (0.94-1.09)	2045

Other female genital	(176)	128	**1.34** (1.09-1.64)	90	101	1.03 (0.85-1.26)	138	229	**1.20** (1.04-1.38)	228

Prostate	(177)	92	1.04 (0.83-1.31)	76	4552	1.03 (1.00-1.06)	7984	4644	1.03 (1.00-1.06)	8060

Testis	(178)	1668	**0.90** (0.84-0.97)	1582	65	0.78 (0.60-1.02)	112	1733	**0.89** (0.83-0.95)	1694

Other male genital	(179)	71	1.15 (0.94-1.42)	60	81	1.22 (1.00-1.48)	98	152	**1.20** (1.05-1.38)	158

Kidney	(180)	663	1.00 (0.90-1.10)	608	716	0.99 (0.89-1.10)	1184	1379	0.99 (0.92-1.06)	1792

Urinary bladder	(181)	489	1.02 (0.90-1.16)	486	1082	0.94 (0.88-1.02)	1964	1571	0.97 (0.91-1.04)	2450

Melanoma	(190)	2789	**0.88** (0.83-0.95)	2952	1310	**0.87** (0.81-0.93)	2232	4099	**0.88** (0.84-0.92)	5184

Squamous cell skin	(191)	412	0.97 (0.84-1.12)	405	638	**0.87** (0.79-0.95)	1309	1050	**0.90** (0.83-0.98)	1714

Eye	(192)	297	1.04 (0.89-1.22)	243	88	0.90 (0.71-1.15)	146	385	1.01 (0.88-1.15)	389

Nervous system	(193)	3730	1.00 (0.95-1.06)	3214	1126	0.99 (0.92-1.07)	1630	4856	1.01 (0.96-1.06)	4844

Thyroid gland	(194)	821	**0.86** (0.79-0.95)	894	156	**0.79** (0.64-0.97)	289	977	**0.85** (0.78-0.92)	1183

Endocrine glands	(195)	1013	0.99 (0.91-1.09)	961	568	**0.87** (0.78-0.98)	952	1581	0.95 (0.89-1.02)	1913

Bone	(196)	500	1.06 (0.94-1.20)	412	37	1.11 (0.69-1.79)	47	537	1.08 (0.95-1.22)	459

Connective tissue	(197)	572	**0.88** (0.78-0.98)	576	161	1.00 (0.82-1.21)	254	733	**0.90** (0.82-0.99)	830

Non-Hodgkin lymphoma	(200, 202)	1330	1.06 (0.97-1.15)	1115	1029	0.99 (0.91-1.08)	1611	2359	1.03 (0.97-1.10)	2726

Hodgkin lymphoma	(201)	1001	0.92 (0.84-1.01)	947	76	1.00 (0.75-1.33)	109	1077	0.93 (0.85-1.01)	1056

Myeloma	(203)	119	0.94 (0.74-1.19)	117	332	1.04 (0.89-1.21)	535	451	1.02 (0.89-1.15)	652

Leukaemia	(204 - 209)	2208	0.98 (0.90-1.06)	1910	709	1.02 (0.93-1.12)	1117	2917	1.00 (0.94-1.07)	3027

Other and unspecified sites	(other)	406	0.91 (0.79-1.04)	398	838	**0.89** (0.80-0.99)	1505	1244	**0.91** (0.84-0.98)	1903

Any site	(any)	29574	**0.97** (0.94-0.99)	27895	29479	0.96 (0.93-1.00)	47948	59053	0.97 (0.95-1.00)	75843

Reference group: first born child. Bold type, 95% CI does not include 1.00. Poisson regression adjusted for age, sex, period, region, socioeconomic status. Ref.: reference, N:number of cases

The association between family size and cancer with singletons as reference is presented in Table 4. Family size was associated with a decreased relative risk for endometrial (RR = 0.76, 99% CI: 0.70-0.84) and testicular cancer, squamous cell skin cancer and melanoma, whereas the opposite was observed for cancer of the nervous system and leukemia (RR = 1.20). For lymphoid leukemia there was a relative risk of 1.30 (95% CI: 1.04-1.61) found for increasing family size (data not shown). In the stratified analysis for the age at diagnosis, the relative risk was increased for stomach cancer (RR = 1.17) and decreased for cervical cancer (RR = 0.82) for individuals above age 50 at diagnosis.

**Table 4:** Relative risks for family size and age at diagnosis

		Age at diagnosis < 50 years			Age at diagnosis ≥ 50 years			All		
			Family size	Ref		Family size	Ref		Family size	Ref
Cancer site	(ICD-7 code)	N	RR (95% CI)	N	N	RR (95% CI)	N	N	RR (95% CI)	N
Upper aero-digestive tract	(140+)	694	1.00 (0.81-1.23)	104	1068	0.96 (0.84-1.09)	316	1762	0.97 (0.87-1.09)	420

Salivary glands	(142)	222	0.85 (0.62-1.17)	38	117	0.87 (0.61-1.24)	38	339	0.87 (0.68-1.10)	76

Esophagus	(150)	85	**0.52** (0.33-0.82)	22	528	0.99 (0.82-1.18)	155	613	0.92 (0.77-1.10)	177

Stomach	(151)	423	0.91 (0.68-1.21)	70	1009	**1.17** (1.02-1.34)	253	1432	1.11 (0.96-1.29)	323

Small intestine	(152)	140	0.76 (0.53-1.09)	29	282	1.14 (0.91-1.44)	71	422	1.03 (0.84-1.27)	100

Colon	(153)	1633	1.01 (0.88-1.17)	233	3213	0.95 (0.89-1.02)	1042	4846	0.97 (0.91-1.03)	1275

Rectum	(154/1541)	632	1.13 (0.78-1.65)	87	2152	0.96 (0.88-1.04)	672	2784	0.98 (0.88-1.09)	759

Anus	(1541)	105	0.93 (0.59-1.48)	17	171	1.03 (0.75-1.41)	47	276	1.01 (0.79-1.30)	64

Liver and gallbladder	(155, 156)	376	1.23 (0.92-1.65)	43	972	0.95 (0.83-1.08)	302	1348	0.99 (0.88-1.11)	345

Pancreas	(157)	231	1.00 (0.72-1.40)	31	1176	0.93 (0.81-1.06)	369	1407	0.94 (0.84-1.05)	400

Nose	(160)	85	1.24 (0.58-2.67)	10	95	1.53 (0.96-2.44)	19	180	1.39 (0.89-2.17)	29

Larynx	(161)	111	1.09 (0.64-1.86)	18	304	1.15 (0.91-1.45)	76	415	1.15 (0.90-1.47)	94

Lung	(162, 163)	926	1.20 (0.97-1.48)	117	4254	0.95 (0.89-1.02)	1290	5180	0.99 (0.93-1.05)	1407

Breast	(170)	8325	0.91 (0.70-1.20)	1506	11473	0.98 (0.95-1.02)	3215	19798	0.96 (0.86-1.08)	4721

Cervix	(171)	2782	0.97 (0.82-1.14)	424	488	**0.82** (0.69-0.98)	158	3270	0.93 (0.82-1.05)	582

Endometrium	(172)	412	**0.67** (0.55-0.81)	122	2145	**0.78** (0.72-0.84)	821	2557	**0.76** (0.71-0.82)	943

Uterus	(173, 174)	258	**1.29** (1.05-1.60)	33	208	0.94 (0.77-1.15)	64	466	1.07 (0.93-1.24)	97

Ovary	(175)	1532	0.98 (0.84-1.16)	238	1542	0.99 (0.89-1.11)	441	3074	0.99 (0.90-1.09)	679

Other female genital	(176)	195	1.32 (0.95-1.83)	23	189	1.02 (0.76-1.38)	50	384	1.14 (0.92-1.40)	73

Prostate	(177)	144	0.85 (0.60-1.21)	24	9489	0.98 (0.95-1.01)	3047	9633	0.98 (0.95-1.02)	3071

Testis	(178)	2857	**0.87** (0.80-0.94)	393	139	0.96 (0.75-1.22)	38	2996	**0.87** (0.81-0.94)	431

Other male genital	(179)	114	1.08 (0.80-1.46)	17	145	1.16 (0.91-1.49)	34	259	1.14 (0.95-1.36)	51

Kidney	(180)	1112	1.01 (0.86-1.19)	159	1460	0.99 (0.88-1.11)	440	2572	0.99 (0.91-1.08)	599

Urinary bladder	(181)	834	1.04 (0.88-1.22)	141	2295	0.94 (0.87-1.02)	751	3129	0.96 (0.89-1.03)	892

Melanoma	(190)	4919	**0.85** (0.78-0.93)	822	2740	0.96 (0.88-1.04)	802	7659	**0.90** (0.84-0.96)	1624

Squamous cell skin	(191)	708	0.98 (0.80-1.21)	109	1433	**0.89** (0.81-0.97)	514	2141	**0.90** (0.82-0.99)	623

Eye	(192)	480	0.97 (0.75-1.25)	60	181	0.96 (0.72-1.28)	53	661	0.97 (0.80-1.18)	113

Nervous system	(193)	6286	1.16 (1.05-1.29)	658	2192	1.06 (0.96-1.16)	564	8478	**1.13** (1.05-1.21)	1222

Thyroid gland	(194)	1496	1.00 (0.87-1.14)	219	356	1.12 (0.89-1.41)	89	1852	1.03 (0.91-1.16)	308

Endocrine glands	(195)	1718	1.00 (0.88-1.14)	256	1189	1.00 (0.88-1.13)	331	2907	1.01 (0.92-1.10)	587

Bone	(196)	821	1.04 (0.84-1.29)	91	69	1.12 (0.71-2.17)	15	890	1.08 (0.89-1.31)	106

Connective tissue	(197)	1013	0.96 (0.81-1.14)	135	318	0.94 (0.76-1.17)	97	1331	0.95 (0.83-1.09)	232

Non-Hodgkin lymphoma	(200, 202)	2163	1.00 (0.85-1.18)	282	2038	0.97 (0.88-1.07)	602	4201	0.98 (0.90-1.07)	884

Hodgkin lymphoma	(201)	1736	0.94 (0.81-1.09)	212	141	0.86 (0.62-1.20)	44	1877	0.93 (0.81-1.07)	256

Myeloma	(203)	211	1.32 (0.89-1.94)	25	660	0.96 (0.82-1.11)	207	871	1.00 (0.87-1.15)	232

Leukaemia	(204 - 209)	3775	**1.30** (1.07-1.59)	343	1429	1.06 (0.95-1.18)	397	5204	**1.20** (1.08-1.33)	740

Other and unspecified sites	(other)	707	1.00 (0.81-1.24)	97	1766	0.90 (0.80-1.03)	577	2473	0.93 (0.84-1.02)	674

Any site	(any)	50261	0.97 (0.94-1.00)	7208	59426	0.99 (0.94-1.04)	18001	109687	0.99 (0.96-1.02)	25209

Reference group: one-child families. Bold type, 95% CI does not include 1.00. Poisson regression adjusted for age, sex, period, region, socioeconomic status. Ref: reference, N: number of cases

The relative risk for cancer of the thyroid gland was marginally significantly decreased for birth order (P for trend = <.0001; data not shown). The relative risk for the second born (RR = 0.87) was slightly higher than for the third or fourth born (RR = 0.82) or higher birth orders (RR = 0.75).

The relative risk for testicular cancer was inversely associated with family size (data not shown). It was decreasing with increasing family size. There was no significant combined effect with birth order even if the trend seemed to show an inverse association. Table 5 shows significantly decreased relative risks with increasing birth order for papillary (RR = 0.81, 95% CI: 0.72-0.92) and follicular thyroid gland tumors (RR = 0.70, 95% CI: 0.52-0.94).

**Table 5:** Relative risks for cancer of the thyroid gland

	All			All		
		Birth order *	Ref		Family size **	Ref
Cancer site	N	RR (95% CI)	N	N	RR (95% CI)	N
Thyroid gland						
papillary	397	**0.81 **(0.72-0.92)	442	736	0.93 (0.77-1.12)	103
follicular	58	**0.70 **(0.52-0.94)	82	116	0.76 (0.50-1.17)	24
medullary	20	0.81 (0.50-1.32)	23	37	0.92 (0.49-1.74)	6
other	31	0.85 (0.59-1.24)	45	64	1.17 (0.69-1.98)	12

Reference group: * = first born child, ** = one-child families. Bold type, 95% CI does not include 1.00. Poisson regression adjusted for age, sex, period, region, socioeconomic status.

Ref: reference, N: number of cases

**Figure 1:**
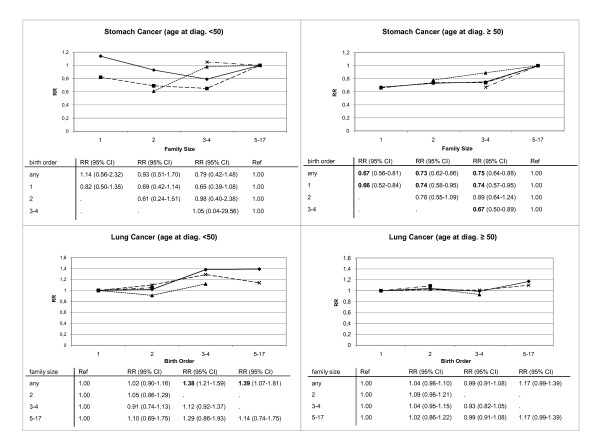
**Relative risks separated for age at diagnosis for lung and stomach cancer**. Relative risks calculated with respect to birth order and family size. Bold type, 95% CI does not include 1.00. Poisson regression adjusted for age, sex, period, region, socioeconomic status

In the separate analysis for the two groups of age at diagnosis (Figure 1), family size was positively associated with stomach cancer for age of diagnosis of at least 50 years. We found highly significant results independent of the birth order (one child families: RR = 0.67; two child families: RR = 0.73; three or four child families: RR = 0.75). Most of the stomach cancer cases occur with an age of diagnosis of at least 50 years and the separate analysis for the younger age group shows no significant associations.

Lung cancer was positively associated with birth order for lower age at diagnosis. The relative risk ranged from 1.02 for second born to 1.38 for third or fourth born offspring to 1.39 for the fifth born. In contrast the relative risk was not significant for different birth order when the age at diagnosis was at least 50 years even if a trend was still noticeable.

**Figure 2:**
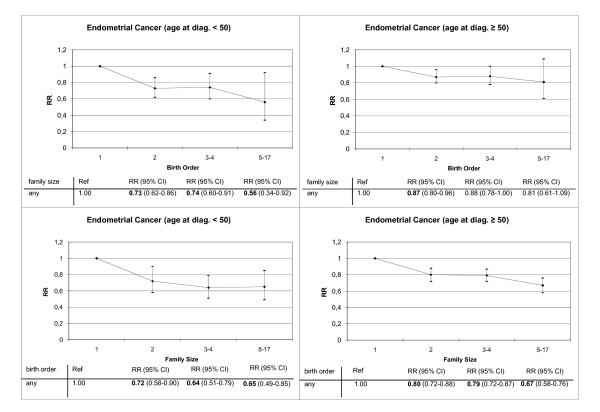
**Relative risks separated for age at diagnosis for endometrial cancer**. Relative risks calculated with respect to birth order and family size. Bold type, 95% CI does not include 1.00. Poisson regression adjusted for age, sex, period, region, socioeconomic status

Figure 2 shows a detailed analysis on endometrial cancer. There was an inverse association of risk and birth order for age at diagnosis below age 50. The relative risk ranges from 0.73 for second born to 0.74 for third or fourth born until 0.56 for at least fifth born with first born children are the reference. For age at diagnosis above 50 years the same trend was observed. Family size was negatively associated with relative risk for endometrial cancer for age at diagnosis below age 50 (one child families: RR = 0.65; two child families: RR = 0.64; three or four child families: 0.72). In contrast, the relative risk in the older age group was negatively associated with family size.

## Discussion

Our results show that there is an effect of family size and birth order on different cancer sites. A significantly decreased risk for testicular cancer by increasing birth order is in line with previously published results [5,6]. Also family size was associated with testicular cancer which has been suggested to be the result of parental subfertility [28].

Family size and birth order showed protective effects for melanoma, in agreement with previous results on socioeconomic status [15,29-31]. We observed a similar association for squamous cell carcinoma, which might be related to the same factors.

Risk for cancer of the nervous system was positively associated with family size which is also supported by previous findings [32]. The number of siblings correlated with the risk for cancer suggesting an infectious etiology [33]. Some subtypes of leukemia as acute lymphoblastic leukemia have their origin in viral or bacterial infection [34,35]. These can be shared easily in large families where all members have closer contact [36].

Family size influenced the risk of stomach cancer. Offspring in families with five or more children had an increased risk which has also been shown in previous studies [19,37]. Helicobacter pylori is an important cause of gastric cancer. The risk of infection is directly associated with sibship size [2,38-40]. As a consequence, members of larger families can easily share some infections.

Risk for lung cancer was increased in large families, most likely because of an association with low socioeconomic status [41,42]. An increasing risk with birth order and family size can clearly be seen for individuals diagnosed below age 50. This might be due to the fact that the effect of birth order and family size is stronger in younger ages, where children still live with their parents and siblings. The effect of birth order and family size decreased from early to later adulthood.

Risk for endometrial cancer decreased with increasing birth order and family size. Family size showed a negative association for endometrial cancer in the group of people diagnosed before 50 years. This is in agreement with already published results reporting obesity as an important risk factor [21,43-45]. Obesity is associated with socioeconomic status [46,47], which may explain the decreased risk of large families with lower socioeconomic status [7]. Low birth order has been shown to be associated with obesity, especially in young women [48]. This might be explained by dietary habits depending on paternal resource.

Negative association between birth order and thyroid gland tumors is in line with already published results [14]. Nevertheless, more biological and epidemiological research is warranted to clarify the exact mechanisms through which higher birth order children have a decreased risk especially for subtypes as papillary and follicular thyroid gland tumors.

There are some limitations to our study. The information of smoking was not registered in our data. We were not able to include the smoking habits in our analysis. This information might have explained the association of lung cancer and birth order in a better way. As well as active, passive smoking can also have an effect on the risk of lung cancer. Lower birth order could lead to more passive smoking if older siblings smoke during the individual's childhood. Active smoking has an effect on the risk of cancer which cannot be taken into account in our analysis. Additionally, the information of obesity was not present in our data which could have helped us to explain effects on the risk of endometrial cancer.

## Conclusion

Our results agree with already published findings on the influence of birth order and family size in melanoma. As these can be explained by the socioeconomic status this could also be an explanation for squamous cell carcinoma which shows an association of risk with birth order and family size. Our findings show that the risk of endometrial cancer is associated with birth order and family size. This can be explained by dietary habits that differ in families with varying number of children. Family size and birth order are associated with different cancer sites not only because of the effect of socioeconomic status. Taken together, our results suggest that the effect of birth order and family size decreases from early to later adulthood for some cancer sites as lung cancer and endometrial cancer.

## Abbreviations

CI: Confidence interval; RR: relative risk; ICD: International Classification of Disease.

## Competing interests

The authors declare that they have no competing interests.

## Authors' contributions

MB carried out the statistical data analysis and wrote the manuscript. MW provided guidance and help in the preparation of the manuscript. HT commented on the manuscript. JS provided the data and commented on the manuscript. KH designed the study and commented on the analysis and the manuscript. All authors have accepted the final manuscript.

## Pre-publication history

The pre-publication history for this paper can be accessed here:

http://www.biomedcentral.com/1471-2407/11/163/prepub
